# Directed Evolution of a Mycobacteriophage

**DOI:** 10.3390/antibiotics8020046

**Published:** 2019-04-25

**Authors:** María Cebriá-Mendoza, Rafael Sanjuán, Pilar Domingo-Calap

**Affiliations:** 1 Institute for Integrative Systems Biology (I2SysBio), Universitat de València-CSIC, 46980 Paterna, Valencia, Spain; maria.c.cebria@uv.es (M.C.-M.); rafael.sanjuan@uv.es (R.S.); 2 Department of Genetics, Universitat de València, 46100 Burjassot, Valencia, Spain

**Keywords:** phage therapy, *Mycobacterium smegmatis*, mycobacteriophages, directed evolution

## Abstract

Bacteriophages represent an alternative strategy to combat pathogenic bacteria. Currently, *Mycobacterium tuberculosis* infections constitute a major public health problem due to extensive antibiotic resistance in some strains. Using a non-pathogenic species of the same genus as an experimental model, *Mycobacterium smegmatis*, here we have set up a basic methodology for mycobacteriophage growth and we have explored directed evolution as a tool for increasing phage infectivity and lytic activity. We demonstrate mycobacteriophage adaptation to its host under different conditions. Directed evolution could be used for the development of future phage therapy applications against mycobacteria.

## 1. Introduction

Antimicrobial resistance is a major global health concern with a current estimated cost of around 700,000 deaths annually which, if not controlled, could raise up to 10 million deaths by 2050 [1]. Tuberculosis, caused by the bacillus *Mycobacterium tuberculosis*, is the 9th leading cause of death worldwide, being responsible for 1.3 million deaths in 2016 [2]. Most of this mortality, though, could be prevented with early diagnosis and appropriate treatment. Some *M. tuberculosis* infections are treated successfully with different antibiotics, but the emergence of antibiotic-resistant strains is a growing source of concern. In addition, *M. tuberculosis* can stay in a dormant state inside alveolar macrophages, a population not targeted by most drugs, making it difficult to eradicate the disease [3]. Under this scenario, there is a need to find potential alternative therapies. One possibility is phage therapy, that is, using bacteriophages (phages) to treat bacterial infections. An interesting property of phages is that some can penetrate into macrophages by phagocytosis [4].

Phages are the most abundant biological entity in the planet with an estimated 10^31^ total particles. Phages were discovered independently in 1915 and 1917 by Twort and d’Hérelle, respectively. Soon after their discovery, d’Hérelle employed phages to treat dysentery in France and cholera in India. Until the Second World War, phage therapy was considered the only therapeutic tool and the only treatment for bacterial diseases, but the discovery of antibiotics and their introduction in the 1940s replaced phage therapy in most countries, particularly in the West. In contrast, in the former Soviet Union phage therapy was used and is still in practice in countries such as Georgia, Russia, and Poland. Differences in language, in the setting-up of clinical trials between Eastern and Western countries, and the emergence of antibiotics has slowed down the progress of phage therapy in the USA and Western Europe for the past 50 years [5]. Currently, however, the antibiotic resistance crisis has led to a reappraisal of phage therapy worldwide.

The field of phage therapy focuses essentially on virulent phages [6]. In addition to killing bacteria rapidly, phages have some advantages over antibiotics, such as their high host specificity, which reduces the damage to other bacteria and hence avoids dysbiosis. Furthermore, phages multiply at the site of infection and only in the presence of their specific bacteria [7]. Based on this, it is expected that phage treatment will require relatively low dosages and treatment frequencies to reach the optimal therapeutic effect [8]. Furthermore, one of the most interesting aspects of phage therapy relates to bacterial resistance, since phages can evolve and overcome resistance [8,9]. Also, directed evolution can help improve phage infectivity, and may be used to reduce resistance emergence rates. Although there are currently no phage therapy products approved as antibacterial drugs for human use in the EU or US, there are ongoing or completed clinical trials [10]. Moreover, the food industry accepts several commercial phage preparations used for biocontrol of bacterial pathogens, which are approved by the FDA, like Listex^TM^ and ListShield^TM^ used to protect food from *Listeria* [11,12].

In this context, we sought to establish proof of concept for the application of phage therapy to mycobacteria. Mycobacteriophages were first isolated in the 1940s using *Mycobacterium smegmatis* as host and are mostly double-stranded DNA, tailed phages belonging to the *Caudovirales* group. Currently, there are 10,454 phages described whose host belongs to *Mycobacterium* genus, of which 1751 are sequenced [13]. Most of these mycobacteriophages belong to the family *Siphoviridae* (with long, flexible, non-contractile tails) and, in a lower proportion, to the family *Myoviridae* (with contractile tails) [14]. Mycobacteriophages have provided a wealth of information on the diversity of phages that infect a common bacterial host. In addition, published sequences suggest a mosaic nature for their genomes, with extensive illegitimate recombination and horizontal gene exchange. Mycobacteriophages have been classified in different groups, called clusters, based on sequence similarity [15]. This previous knowledge has provided a variety of tools that can be employed to study mycobacterial genetics and, also, to establish new strategies to control, diagnose and treat diseases caused by mycobacteria [16].

Our main goal here was to demonstrate that evolution under controlled conditions can help us obtain more infective mycobacteriophages. To achieve this aim, we evolved a phage by serial passages under different conditions using *M. smegmatis* as model host. *M. smegmatis* is a non-pathogenic bacterium with a faster life cycle than other *Mycobacterium* species [17], thus offering a good system to explore and set up directed evolution protocols.

## 2. Results

### 2.1. Directed Evolution

**Figure 1:**
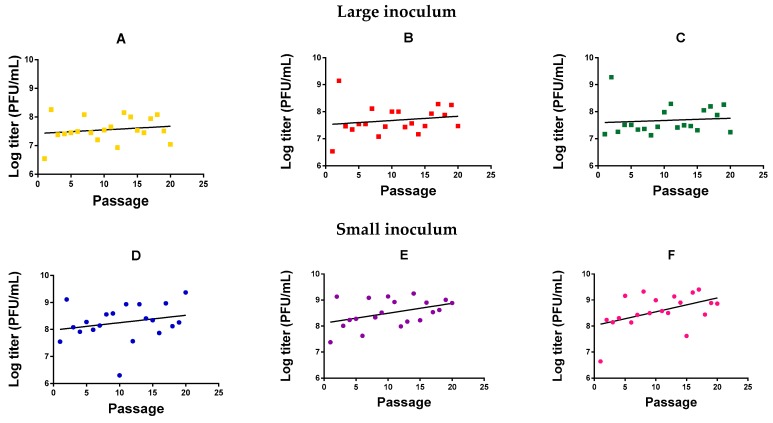
Log-titer reached after each passage of mycobacteriophage evolution. Large inoculum: (**A**) Yellow, (**B**) red, (**C**) green. Small inoculum: (**D**) Blue, (**E**) purple, (**F**) pink. Dots: Experimental points. Line: Linear regression.

We evolved six independent lines (evolution replicates) of *M. smegmatis bacteriophage* (American Type Culture Collection, ATCC^®^ 11759-B1^TM^) in *M. smegmatis* for 20 serial transfers (passages) in semi-solidified medium, three (Figure 1A–C) using a large phage inoculum per passage (10^5^ plaque forming units, PFU) and three (Figure 1D–F) using a small phage inoculum (10^2^ PFU). Independent of passage number, we observed that the phage reached higher titers with a small inoculum (1.28 ± 0.53 × 10^9^ PFU/mL) than with a high inoculum treatment (1.94 ± 0.54 × 10^7^ PFU/mL; *t*-test using log-transformed titers: *p* = 0.001). This probably indicates that the large inoculum exhausted the cell population rapidly, whereas a smaller inoculum allowed cells to proliferate for longer, increasing the number of susceptible host cells and, thus, the total amount of viral progeny produced. For the small-inoculum lines, a linear model using the evolution replicate (Figure 1D–F) as a random factor and passage number as a covariate showed that the log-titer increased significantly with passage number (*F* = 9.071, *p* = 0.004; Figure 1). In contrast, in phages evolved using a large inoculum size, we could not detect an effect of passage number on log-titer (*F* = 1.131, *p* = 0.292; Figure 1A–C).

### 2.2. Analysis of Mycobacteriophage Growth Rate and Stability

**Figure 2:**
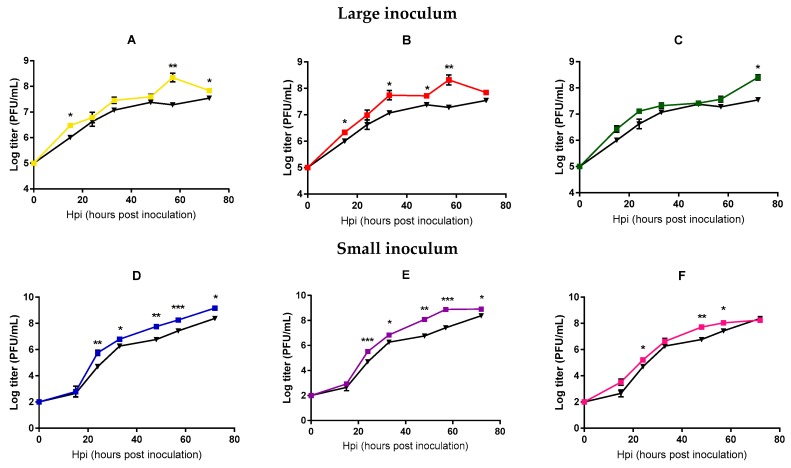
Mycobacteriophages growth curves of the founder and evolved lines. Founder: Black. Large inoculum: (**A**) Yellow, (**B**) red, (**C**) green. Small inoculum: (**D**) Blue, (**E**) purple, (**F**) pink. Dots show the average titer at each time point and error bars indicate the SEM (*n* = 3). *: *p* < 0.05, **: *p* < 0.005, ***: *p* < 0.0005.

In order to more directly test whether viral fitness changed significantly after 20 passages, we performed standard growth curves in triplicate, in which we compared the founder phage and each of the evolved phages in the same experimental block. Each line was tested using the same inoculum size employed during the evolution experiment. Significant differences between the founder and the evolved lines were found at intermediate and late points (*t*-tests, *p* < 0.05, Figure 2), suggesting an adaptation of all the evolved lines under our different conditions, although less marked for those lines evolved under large inoculum conditions.

**Figure 3:**
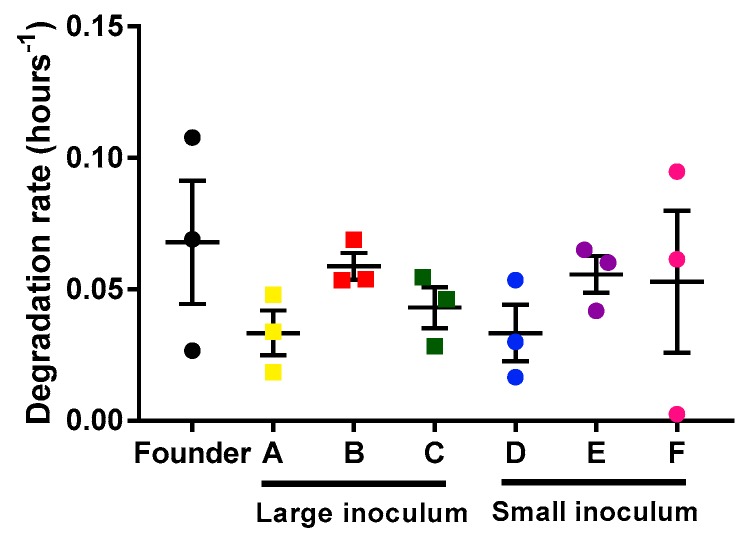
Mycobacteriophages degradation rates of the founder and evolved lines. Founder: Black. Large inoculum: (**A**) Yellow, (**B**) red, (**C**) green. Small inoculum: (**D**) Blue, (**E**) purple, (**F**) pink. Dots show the experimental replicates, and error bars indicate the SEM (*n* = 3).

In principle, differences in the population growth rate of the phage could be due to the faster infection rate or to the slower degradation rate of the phages. We determined the phage’s degradation by measuring the decrease in viral titer as a function of time on the infection medium (in the absence of bacteria). For each lineage (founder and evolved lines), three replicates were done to estimate the degradation rate (Figure 3). Our results showed no differences in degradation rate between the different lines (unpaired *t*-tests, *p* > 0.05). Hence, the observed acceleration of phage growth was driven by a more rapid infection, which in turn could be due to faster adsorption, faster replication, or increase phage yield per cell.

### 2.3. Analysis of Bacterial Lysis Efficiency

**Figure 4:**
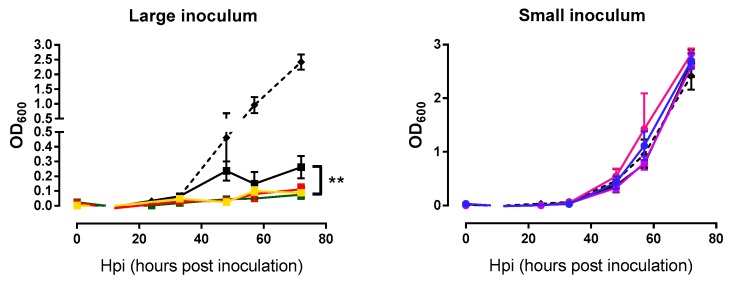
Bacterial growth curves of the founder and evolved lines. Founder: Black. Large inoculum: (**A**) Yellow, (**B**) red, (**C**) green. Small inoculum: (**D**) Blue, (**E**) purple, (**F**) pink. Dots show the average OD_600_ at each time point and error bars indicate the SEM (*n* = 3). Control without phage infection in dashed line. **: *p* < 0.005.

In addition to examining the phage growth rate, we were interested in assessing whether the evolved lines killed *M. smegmatis* more efficiently than the founder phage. Measurements of bacterial densities at the same time points used above for the phage growth curves were done in triplicate. Again, each phage line was tested using the same inoculum size employed during passages. For large inoculum condition, evolved phages were able to lyse bacteria more efficiently than the founder (nested ANOVA: Founder vs. evolved *p* = 0.007, among lines *p* = 0.082; Figure 4). In contrast, these assays were not capable of detecting differences in lysis activity under small-inoculum conditions (nested ANOVA: Founder vs. evolved *p* = 0.715, among lines *p* = 0.174; Figure 4) because small inocula did not produce an appreciable change in bacterial density, independent of the phage used. In other words, with large inocula phages were able to reach the majority of bacterial cells growing in the culture dish, whereas, small inocula gave rise to few, isolated plaques which had little effect on overall bacterial counts.

**Figure 5:**
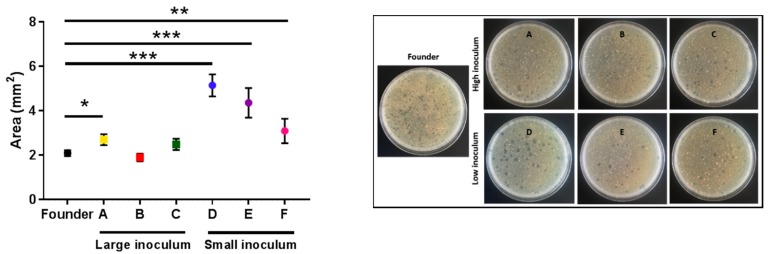
The average area of the plaques of the founder and evolved lines. Founder: Black. Large inoculum: (**A**) Yellow, (**B**) red, (**C**) green. Small inoculum: (**D**) Blue, (**E**) purple, (**F**) pink. Dots show the average area for each line and error bars indicate the SEM (*n* = 3). *: *p* < 0.05, **: *p* < 0.005, ***: *p* < 0.0005. Right panel: Representative images of the plates for each line.

To better examine the killing capacity of phage evolved under the small-inoculum regime, we plated all the evolved lines in triplicate to determine differences in the surface area of the plaques using image analysis. Clear differences between the plaque sizes of lines evolved under the small inoculum regime and the founder were detected (*t*-tests: D: *p* < 0.001; E: *p* < 0.001; F: *p* = 0.009; Figure 5). A tendency towards increased plaque size was also observed for lines evolved under the large inoculum regime, albeit the effect was less evident than for small-inoculum lines and reached significance only for one of the evolved lines (*t*-tests: A: *p* = 0.019; B: *p* = 0.408; C: *p* = 0.134; Figure 5).

## 3. Discussion

Directed evolution is a powerful approach for the optimization of different biological processes. The evolution of phage and bacteria has been investigated experimentally in many previous studies [18,19,20,21,22]. However, the experimental evolution of large double-stranded DNA viruses has been less extensively explored, probably because of their lower mutation rates [23], which should slow down evolutionary processes compared to RNA viruses. Yet, some DNA phages have been shown to evolve at rates close to those of RNA phages in the laboratory [18]. We note, though, that there is little or no previous work exploring the evolution of mycobacteriophages under controlled laboratory conditions. Here, we found that phages evolved under our experimental conditions improved fitness as indicated by different analyses. Adaptation has been observed in all the evolved lines (large and small inoculum), demonstrating that the ability to kill bacteria increases after 20 serial passages. In general, higher inoculum sizes achieve higher effective population sizes [19], and increasing the population size typically improves adaptation because this increases allelic diversity and strengthens the efficacy of natural selection relative to random genetic drift [24,25]. However, the dynamics of the host population is also an important factor. If the inoculum is large, the population of host cells can become exhausted rapidly, leaving no resources for further viral replication [26]. This shortens the number of viral generations per passage, potentially slowing adaptation down on an absolute time basis. Clearly, our small-inoculum evolution regime selected for larger plaque sizes than the large-inoculum regime. This increase in plaque size indicates faster spread and more efficient lysis, both of which are interesting properties for phage therapy. Phages evolved under the large-inoculum regime showed increased lysis activity as determined by OD_600_ measurements, but plaque size measurements revealed little or no improvement in spread ability. Based on this and, since in real treatment settings it is likely that the initial ratio of phage to target bacteria is small, we suggest that directed evolution protocols aimed at increasing the ability of a phage to lyse bacterial populations should be preferably performed under small-inoculum conditions.

Directed evolution can be carried out in liquid or in semi-solidified media. Liquid media combined with shaking allows for more efficient propagation of the phage, whilst semi-solidified media impose spread constraints due to the spatial structure. We believe that the latter approach provides a more realistic scenario for phage adaptation and, hence, may select for better phages. Additionally, a liquid culture of mycobacteria is generally difficult because mycolic acids promote cellular aggregation, generating clumps. Adding detergents to the culture can avoid clumps, but these will typically inhibit phage infectivity. For these reasons, we performed our serial transfers in culture dishes instead of liquid-culture tubes. Phage diffusion is limited in semi-solidified media and is dependent on virion morphology and size. In most cases, phage progeny from one cell will infect neighbor cells only, imposing selection pressures that probably differ from those of well-mixed populations. Mycobacteriophages should be able to increase fitness in many different ways, including improvement in the attachment to cell receptors, increased burst size, reduced lysis time, or faster replication, among others, and the outcome may depend on medium viscosity. A better understanding of the molecular mechanisms responsible for adaptation would be obtained by sequencing the evolved lines. Yet, this can be difficult due to the relatively large genome of mycobacteriophages compared to other viruses and the large number of genes with unknown functions.

Bacteria can evolve phage resistance fast by different mechanisms, including loss of the receptor or activation of the CRISPR system [27,28]. Antagonistic coevolution between phage and bacteria implies ongoing selection of host resistance and resistance breaking, which results in changes in phage and host genomes across evolutionary time [29]. Previous works have shown that infection efficiency improves in comparison with a wild-type phage when the coevolution process is performed using preadapted phages (phages which have been subjected to evolutionary passages) [19,20]. This might be an alternative strategy for achieving resistance breaking and for obtaining in the laboratory a phage population capable of preventing the emergence of resistant bacteria during treatment.

Our work provides proof of concept for the use of directed evolution in a mycobacteriophage. Despite the fact that phages are highly specific to their hosts, some mycobacteriophages can infect both *M. tuberculosis* and *M. smegmatis* [30]. Thus, it is possible that our results are applicable to *M. tuberculosis*. However, additional experiments should be done to explore this possibility. 

## 4. Materials and Methods 

### 4.1. Bacterial Strains and Culture Conditions

We used *M. smegmatis* Δ1366 (*M. smegmatis*) [31], kindly provided by Dr. José Luis García (CSIC). This strain is a clone of mc^2^155 which has the *MSMEG_1366* gene deleted (ABC transporter, ATP-binding protein) and has a plasmid encoding kanamycin resistance and Venus fluorescent protein. *M. smegmatis* was grown in Lysogeny Broth (LB) medium at 37 °C with shaking (250 rpm) in the case of liquid media [16]. LB was supplemented with kanamycin 20 μg·mL^−1^, as well as with 5 mM CaCl_2_ to improve phage growth. For growing bacteria in semi-solidified media, 1.5% of agar was added to LB and kanamycin was omitted. In order to preserve bacteria, glycerol 20% (*v*/*v*) was added to bacterial cultures, which were stored at −70 °C. A stock of non-evolved bacteria was done in a first step. For this purpose, a culture of *M. smegmatis* in supplemented LB was incubated until OD_600_ = 0.3 was reached. The culture was divided into 50 mL tubes and chilled on ice. These tubes were centrifuged at 2000× *g* for 15 min at 4 °C. The supernatant was discarded and the pellet was resuspended in ca. 30 mL of LB with 15% of glycerol to obtain a 40× concentrated stock. The culture was split into 250 μL aliquots that were flash-frozen in liquid nitrogen and stored at −70 °C. 

### 4.2. Mycobacteriophage

The *M. smegmatis bacteriophage* was obtained from ATCC (reference ATCC^®^ 11759B1^TM^). Phage buffer (Tris-HCl pH 7.5, 10 mM MgSO_4_, 5 mM CaCl_2_ and 68.5 mM NaCl) [13] was used for phage dilutions, phage recovery and phage storage. For phage propagation, *M. smegmatis* was grown to an OD_600_ of 0.3 in supplemented LB. We mixed 100 μL of *M. smegmatis* culture with 100 μL of phage. This mix was incubated without shaking for 15 min at room temperature to allow for phage adsorption. The infection was inoculated in 2 mL of supplemented LB and was incubated for 48 h at 37 °C. After that, cultures were centrifuged at 16,000 × *g* for 1 min, the pellet was discarded and the supernatant was aliquoted and stored at −70 °C. For phage titration, serial dilutions of each aliquot were prepared and 100 μL of these dilutions were employed to infect 10^7^ CFU of *M. smegmatis.* Titration was done onto LB plates with top agar (LB with 0.7% agar) and plates were incubated for 40 h at 37 °C. After this, plaques were counted to determine titers.

### 4.3. Evolution

The indicated amount of PFU was used to inoculate 10^7^ CFU of non-evolved *M. smegmatis* obtained from our frozen stock (see above) at an initial OD_600_ of 0.3. Two different conditions were tested: Large inoculum (10^5^ PFU) and small inoculum (10^2^ PFU). Twenty serial passages from plate to plate were performed, controlling the initial inoculum (10^5^ PFU or 10^2^ PFU) for each replicate at each passage. For this, after each passage, phages were titrated by the plaque assay. For each condition, three evolution replicates (lines) were established. Inoculations were carried with 100 µL of phage suspension onto LB plates with top agar and incubated for 40 h at 37 °C. To collect phages, 5 mL of phage buffer was added to the plates and incubated for 2 h at 37 °C [32]. Then, 2 mL of this phage buffer were collected and cells were removed by centrifugation (16,000× *g* for 1 min). The supernatant was aliquoted and stored at −70 °C. One aliquot of each condition was used for the next passage. 

### 4.4. Growth Curves

Infections were started with 10^5^ PFUs in the case of phages evolved using a high inoculum size and with 10^2^ PFUs in the case of phages evolved with a low inoculum size, that is, under the same conditions used for evolution. The founder phage was included in all assays. For each time tested, we performed three replicates per line. Infections were performed as above onto LB plates with top agar and incubated at 37 °C for different times (0 hpi, 15 hpi, 24 hpi, 33 hpi, 46 hpi, 57 hpi and 72 hpi). Then, plates were flooded with 5 mL of phage buffer and incubated for 2 h at 37 °C. The buffer (2 mL) was collected and OD_600_ measures were done for each replicate at each time point. Afterward, samples were centrifuged (16,000× *g* for 1 min) to remove cells, and supernatants were aliquoted, stored at −70 °C and titrated.

### 4.5. Mycobacteriophage Degradation Rates

Founder and evolved lines were used to estimate the mycobacteriophage degradation rate. For this, 10^4^ PFUs were added to 3.5 mL of top agar in the absence of bacteria and were plated in semi-solidified agar plates and titrated at 0 hpi, 24 hpi, 48 hpi, and 72 hpi, as described previously.

### 4.6. Plaque Sizes

Estimates of plaque sizes for the evolved and ancestral lines were done in triplicate. Plating was done by carrying out plaque assays with an estimated 100 PFUs. Plaque size was determined at 72 hpi and was calculated by image analysis using ImageJ software (National Institutes of Health, Bethesda, MD, USA).

## 5. Conclusions

The present work is a step forward in the use of directed evolution as an optimization tool in mycobacteriophages. We have shown that evolved phages exhibit improved lytic activity compared with the founder phage under our experimental conditions. Our results might help in the development of future treatments against pathogenic and multi-resistant *Mycobacterium tuberculosis* strains, and suggest phage therapy as a potential alternative to the conventional antibiotics.
